# Risk and Protective Factors for Obstructive Sleep Apnea Syndrome Throughout Lifespan: From Pregnancy to Adolescence

**DOI:** 10.3390/children12020216

**Published:** 2025-02-12

**Authors:** Duilio Petrongari, Francesca Ciarelli, Paola Di Filippo, Armando Di Ludovico, Sabrina Di Pillo, Francesco Chiarelli, Giulia Maria Pellegrino, Giuseppe Francesco Sferrazza Papa, Luana Nosetti, Marina Attanasi

**Affiliations:** 1 Department of Pediatrics, Pediatric Allergy and Pulmonology Unit, University of Chieti-Pescara, Via Dei Vestini 5, 66100 Chieti, Italy; duilio.petrongari@studenti.unich.it (D.P.); francesca.ciarelli@studenti.unich.it (F.C.); paola.difilippo@asl2abruzzo.it (P.D.F.); armando.diludovico@studenti.unich.it (A.D.L.); sabrina.dipillo@asl2abruzzo.it (S.D.P.); chiarelli@unich.it (F.C.); 2 Department of Neurorehabilitation Sciences, Casa di Cura Igea, 20144 Milan, Italy; g.pellegrino@casadicuraigea.it (G.M.P.); giuseppe.sferrazza@unikore.it (G.F.S.P.); 3 Department of Pediatrics, Pediatric Sleep Disorders Center, F. Del Ponte Hospital, Insubria University, 21100 Varese, Italy; luana.nosetti@uninsubria.it

**Keywords:** obstructive sleep apnea syndrome, obstructive sleep disorders, sleep disorders, adenotonsillar hypertrophy, obesity, children, adolescence, prenatal sleep

## Abstract

Background: Obstructive sleep apnea syndrome (OSAS) in children is indeed a significant and often underdiagnosed condition. The risk factors for OSAS vary across different stages of life. Objectives: Identifying risk factors early can help in taking preventive measures to reduce the likelihood of developing OSAS, and different life stages may require different interventions. Results: During pregnancy, maternal factors such as obesity, smoking, and genetic predispositions can increase the risk of OSAS, while breastfeeding serves as a protective factor. For children aged 2 to 12, adenotonsillar hypertrophy is the primary cause of airway narrowing, with other contributing factors including obesity, craniofacial abnormalities, and increased nasal resistance. In adolescence, obesity and craniofacial abnormalities remain the main risk factors. Conclusions: By reviewing and understanding these risk factors, healthcare providers can offer more personalized and effective care, ultimately leading to better health outcomes for individuals at all stages of life.

## 1. Introduction

Sleep-disordered breathing (SDB) encompasses a wide range of disorders characterized by a variable obstruction of the upper airways and altered gas exchange during sleep. These disorders are due to several factors facilitating increased upper airway resistance and larynx collapse [1,2]. SDBs are classified into simple snoring, Upper Airways Resistance Syndrome, obstructive hypoventilation, and obstructive sleep apnea syndrome (OSAS) [3]. OSAS refers to a recurrent partial or complete obstruction of the upper airway, which causes brief episodes of interrupted breathing (apnea) or markedly reduced airflow (hypopnea) during sleep, resulting in increased respiratory effort, sleep fragmentation, cerebral arousal, and oxyhemoglobin desaturation [4,5]. The third edition of the International Classification of Sleep Disorders defines OSAS as a condition characterized by an obstructive respiratory disturbance index (RDI) observed at polysomnography (PSG) ≥ 5 events/hour associated with typical symptomatology or an RDI ≥ 15 events/hour even if asymptomatic. Changes in respiratory flow during OSAS must be associated with either a 3% oxygen desaturation or arousal, although an alternative definition requires a 4% desaturation not taking arousal into account [6]. An Apnea–Hypopnea Index (AHI) between 5 and 15 diagnoses mild OSAS, between 15 and 30 moderate, and >30 severe disease [1]. In young children (1 to 23 months old) with risk factors for OSAS, mild OSAS is frequently diagnosed with obstructive AHI > 1–5 episodes per hour, moderate OSAS with obstructive AHI > 5–10, and severe OSAS with obstructive AHI >10 [7].

OSAS affects 1.2 to 5.7% of children with a peak prevalence between 2 and 8 years of age, mirroring the peak of maximum development of tonsillar and adenoid tissue [8]. The pathophysiological mechanisms of pediatric OSAS are not yet fully understood. However, factors influencing airway collapsibility, or producing anatomical narrowing of the upper airways, or a combination of both are recognized as underlying mechanisms [9]. Obstructive causes are observed in more than 95% of cases, while less than 5% of cases are due to central nervous system disorders (cerebral palsy, myotonic dystrophy, and dysautonomia) [4].

The main cause of airway narrowing in OSAS is adenotonsillar hypertrophy (ATH). Other contributing factors include obesity, craniofacial abnormalities, syndromic and non-syndromic alterations (retrognathia, micrognathia, hypoplasia, and mild face hypoplasia), macroglossia, increased nasal resistance, and lingual tonsil hypertrophy [8]. Childhood OSAS is now recognized as a major public health problem. It is associated with significant morbidity and increased healthcare referral, particularly in the case of obesity [10]. OSAS can cause cardiovascular, metabolic, learning disabilities, behavioral problems and growth delays in children [4]. The aim of our narrative review was to analyze the risk factors of OSAS in children to better understand its underlying physiopathological mechanisms and to perform a tailored phenotype-based approach to diagnosis and therapy.

## 2. Materials and Methods

**Figure 1:**
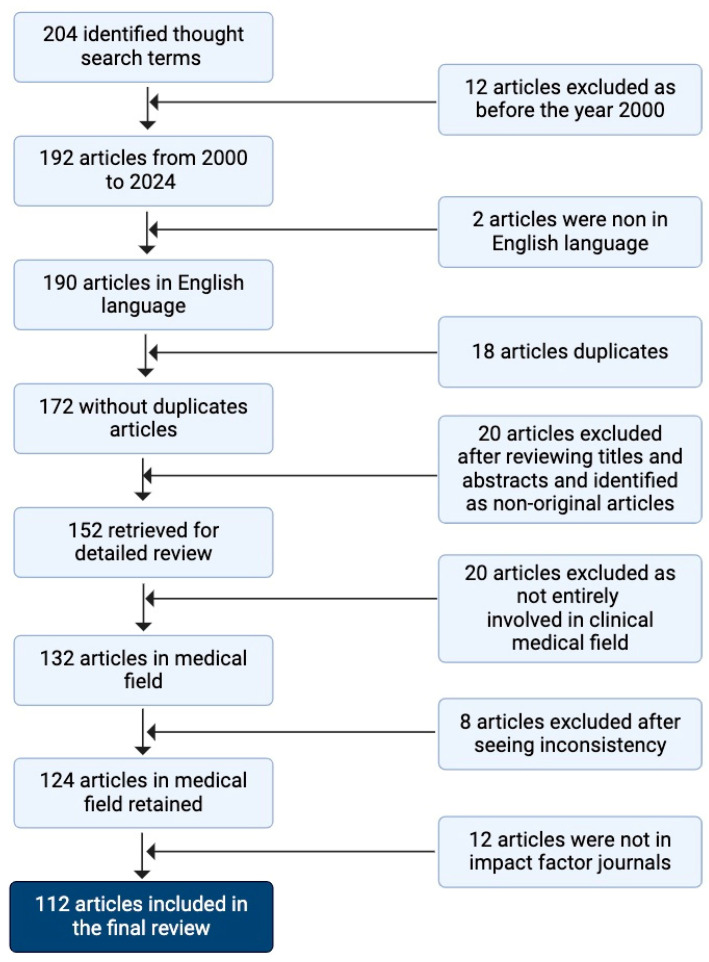
Flowchart of the selection of reviewed articles.

A strategic search of original published articles in this field from 2000 to 2024 was performed using PubMed and Google Scholar. The following search terms and logic were employed for the introduction and general paragraphs on predictors for obstructive sleep apnea syndrome in children: “sleep-disordered breathing” OR “sleep disorder” OR “obstructive sleep disorder” OR “sleep study” OR “noisy breathing” OR “polysomnography” OR “habitual snoring” AND “pediatric” OR “young” OR “infant” OR “children” OR “adolescents”. Additional search terms were adopted for each specific paragraphs: for the predictors of OSAS during pregnancy, the first two years of life and preschool age “pregnancy outcome” OR “gestational hypertension” OR “prenatal health” OR “early-life predictors” OR “maternal health”; for the predictors of OSAS during school age paragraph, “adenotonsillar hyperthrophy” OR “obesity” OR “atopy” OR “air pollutants” OR “allergic rhinitis” OR “family history” OR “male gender”; and for the predictors of OSAS during adolescence paragraph, “adenotonsillar volume” OR “neuromotor factors” OR “smoking” OR “personalized medicine” OR “biomarkers”. Further studies were obtained through the reference of some papers. Articles were selected according to their titles and abstract, using eligibility criteria. The inclusion criteria were as follows: being original research articles written in English in peer-reviewed journals; those that were involved in clinical medicine; studies involving only a pediatric study population (age range 0–18 years) or both children and adults; and these types of studies: narrative and systematic reviews, longitudinal retrospective and prospective studies, and randomized control trials. Data were collected and stored in a database. Then, data were checked to find discrepancies between the two reviewers. The final reference list was developed on the basis of originality and relevance to the broader scope of this review. The flowchart of the selection of reviewed articles is shown in the Figure 1.

## 3. Risk Factors

There are studies indicating OSAS has been associated with several controversial risk factors, such as allergic rhinitis (AR) [11], premature birth [12], parental smoking [13], low socio-economic status [14], and African American race [13,15]. Importantly, different potential predictors are associated with the risk of developing OSAS in children of different ages.

### 3.1. Predictors of OSAS During Pregnancy, the First Two Years of Life, and Preschool Age

**Figure 2:**
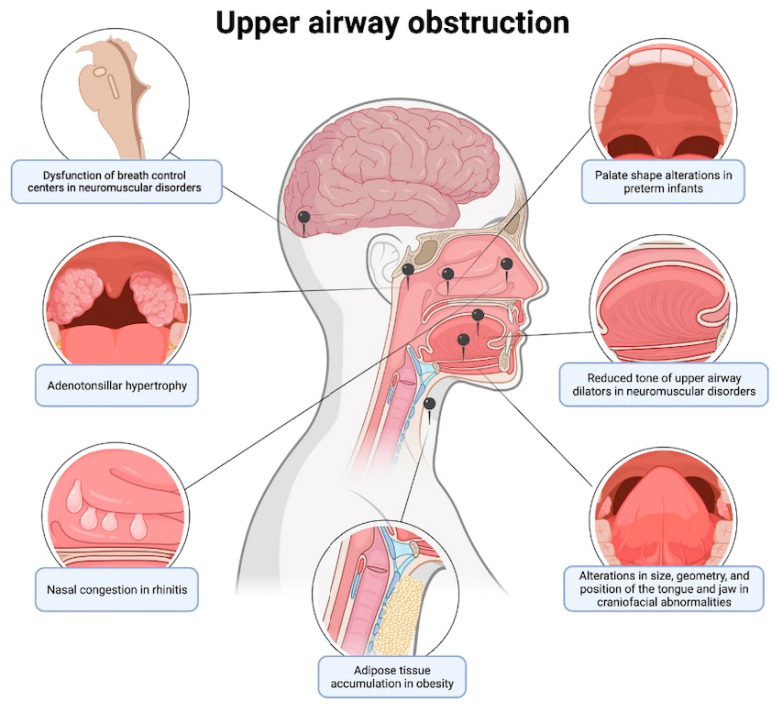
Anatomical and physiological insights into upper airway obstruction. This figure provides an overview of the multifaceted causes leading to upper airway obstruction, highlighting anatomical contributors such as adenotonsillar hypertrophy and palatal shape changes in preterm infants, as well as physiological factors including reduced upper airway tone and compromised neuromuscular disorders.

There are several predictor factors that contribute to the development of OSAS. Some are specific to the prenatal period such as maternal factors during pregnancy [13], genetic polymorphisms [4], gender [16], ethnicity [8], and craniofacial abnormalities [17]. Crump et al. [13] carried out cohort study 4,195,249 with patients diagnosed with SDB with the aim of estimating the risk of developing SDB according to gestational age at birth. High maternal BMI and smoking during pregnancy increased the risk of developing OSAS, while maternal age appears to be inversely proportional to the risk. Several studies have highlighted the role of genetic factors in the development of OSAS. Numerous genetic polymorphisms appear to have a high association with the development of OSAS and associated cognitive dysfunction in childhood [4]. Gender too plays a relevant risk factor, with a higher prevalence in males [16,17,18]; a randomized study including 6447 primary school children showed an OSAS incidence of 5.8% in males and of 3.8% in females [19]. The gender discrepancy is probably due to longer airways in post-pubertal males, with a major tendency to collapse [20,21] and differences in neurochemical control mechanisms and the arousal response [22]. Another risk factor for developing OSAS is African-American ethnicity [8,15]. African-American children have a 4–6 times greater risk of developing OSAS and severe nocturnal hypoxemia than Caucasian children [23,24]. A prospective study of children with OSAS candidates for adenotonsillectomy (AT) identified an average AHI value 20% higher in children of African American descent than children of other ethnicities [24]. The predisposition of those children appears to be linked to differences in airway anatomy, neuromotor control of the pharynx, and other genetic or environmental factors unknown to date [23]. Importantly, anatomical sites, such as oropharynx, nasopharynx, and hypopharynx (Figure 2) are critical in maintaining an open airway during sleep, and any abnormalities or obstructions in these regions can contribute to OSAS. Other risk factors include a combination of craniofacial anatomical variants, muscle weakness, and dysfunction in the central control of breathing. Common abnormalities associated with OSAS include the following: mild facial hypoplasia, retro/micrognathia, naso-septal obstruction, macroglossia, steep mandibular plane, vertical craniofacial growth direction, and class II malocclusion [4,23]. The retropalatal region is the most frequent site of obstruction in children [25]. The prevalence of OSAS is 48–83% in children with micrognathia and glossoptosis [26]. Furthermore, these patients abnormalities show persistent OSAS even after the removal of adenoids, tonsils, or both [4]. Certain genetic diseases are closely correlated with the development of OSAS, due to typical craniofacial malformations which act on the soft tissue of the upper airways favoring the development of OSAS [27]. The incidence of OSAS is particularly high in children with Down syndrome (74%) [28] as a result of ATH, which reduces the airway size due to mild facial hypoplasia and a small mandible [29], Prader Willi syndrome (79.9%) [30], and achondroplasia (59%) [31]. Craniofacial abnormalities are also the main contributors to a high incidence of OSAS in other genetic disorders, such as mucopolysaccharidosis [32], or in Pierre–Robin, Noonan, and Ellis–van Creveld syndromes, and other syndromes characterized by craniofacial dysostosis or micrognathia [33]. Screening for OSA and treatment in children with craniofacial alterations is a challenge. Screening instruments for OSA may be unreliable in otherwise healthy children and in general have not been validated in the population, where cognitive and hearing deficits may alter the results of such instruments [17].

Gestational age may also play an important role in the development of respiratory problems [34]. Particularly, infants born prematurely (<37 weeks gestational age) have a 3- to 5-fold risk of developing OSAS during childhood than children born at term [12]. School-aged children with a positive history of prematurity had a higher prevalence of OSAS (9.6%) diagnosed with PSG than controls (1–4%) [35]. Similarly, Rosen et al. [36] observed an almost 3-fold increased risk of OSAS in 8–11-year-old children born preterm compared with those born at term. The risk of developing SDB in preterm infants is highest during early childhood (0–9 years), then decreases slightly during late childhood and adolescence (10–19 years) before strengthening again in adulthood (20–29 years). Several factors may explain these findings. Facial asymmetry or an elongated head shape is common in these patients, altering the size and growth of the upper airway. Additionally, endotracheal intubation and application of naso-gastric tubes may alter the shape of the palate and growth of the airways. Muscular hypotonia is also common in those patients [37]. Contrarily, maternal breastfeeding appears to be a protective factor against SDB [38,39,40].

The role of respiratory infections as risk factors for the development of SDB in childhood is still debated; viral-type infections probably are the main culprits. Snow et al. [41] observed that children with bronchiolitis caused by respiratory syncytial virus (RSV) showed a significantly higher obstructive apnea/hypopnea index than controls. Additionally, in children with a previous history of bronchiolitis, respiratory arousal indices were significantly higher than controls. Goldbart et al. [42] evaluated the possibility that RSV infection could be associated with OSAS, promoting the proliferation of adenoid and tonsillar tissue through the upregulation of the nerve growth factor (NGF) receptor neurokinin 1 and substance P. Other viruses, such as rhinoviruses, could similarly exhibit a particular trophism towards adenoid tissue [43].

Several studies found an association of brief, resolved, unexplained events (BRUEs)/apparently life-threatening events (ALTEs), with the presence or subsequent development of OSAS [44]. Harrington et al. [45] investigated cardiorespiratory control in infants with ALTE compared with age-matched controls. The authors observed that none of the controls had abnormalities in nocturnal breathing on a polysomnography while 5 out of 10 infants with ALTE presented two or more obstructive apneas during sleep. The European Respiratory Society states that all patients with a previous history of BRUE are at risk of developing OSAS [7]. However, further larger and well-designed studies are needed to assess the possible appearance of SDB or PSG abnormalities during the follow-up of infants with BRUE.

Sleep-disordered breathing in children with neuromuscular diseases is more prevalent compared to the general population and often manifests as sleep-related hypoventilation, sleep-related hypoxemia, obstructive sleep apnea, central sleep apnea, and/or disordered control of breathing [46]. Cerebral palsy, congenital muscular dystrophy, myotonic dystrophy, mitochondropathies, or spinal cord injury contribute to OSAS development leading to an altered muscle tone [4,7]. Patients with neuromuscular disorders present increased upper airway resistance due to hypotonia of the upper airways and a lower residual lung capacity because of a decreased intercostal muscle tone, sometimes in addition to diaphragm weakness. These conditions are amplified during the Rapid Eye Movement (REM) phase of sleep where inhalation depends mainly on the diaphragm and a marked reduction in muscle tone is present [47]. The entity of SBD depends on the course of the neuromuscular disease [46]. Chacko et al. [48] carried out a prospective cohort study of children with spinal muscle atrophy (SMA) types 1 to 3 and <19 years who showed increased respiratory events in REM sleep than non-REM sleep. Both central and obstructive events (central > obstructive) were more common in SMA type 1. Suresh et al. [49] observed that patients with Duchenne disease aged 8 years with preserved respiratory muscle function more frequently presented OSAS than older patients (mean age 13 years) with major muscle weakness who showed more episodes of hypoventilation. Myelomeningocele, Arnold–Chiari malformation, and brain lesions due to trauma, tumors, surgery, or radiotherapy predispose one to the development of OSAS [10] as they can damage the respiratory control center located in the brainstem. Therefore, they show a predominance of central apneas compared with obstructive events [46]. Children with neuromuscular disorders should be evaluated for SBD, as it can contribute to a further deterioration in quality of life (QoL) by worsening their respiratory status and a significantly increasing morbidity of mortality [50,51].

### 3.2. Predictors of OSAS During School Age

Specific predictor factors for developing OSAS are described for school-aged children such as adenotonsillar hypertrophy (ATH) [15], atopic predisposition [52], exposure to environmental pollutants [53], and socio-economic status [54].

Adenotonsillar hypertrophy is the most important risk factor for developing OSAS in children [15], especially between the ages 2 and 8, when there is the maximum development of tonsillar and adenoid tissue [8]. The first-line treatment for children with moderate to severe OSAS due to ATH is adenotonsillectomy, a safe procedure in 93% of cases with a 75% of success rate [55]. In a meta-analysis including 14 studies, Brietzke et al. [56] showed that the removal of adenoids and tonsils led to the normalization of the polysomnographic results in 83% of children undergoing this procedure. Lundkvist et al. [57], analyzing data from 3 million patients aged 0–18 years, observed that children of parents diagnosed with OSAS, had a significantly increased incidence of having adenotonsillar hypertrophy. Indeed, OSAS incidence rates in patients with at least one affected parent were significantly higher than in general population [58,59]. The familial predisposition appears to be related to polymorphisms in the synthesis of cysteinyl leukotrienes involved in the overproduction of pharyngeal lymphoid tissue [60]. ATH is a predictor of severe OSA, and children with this condition should be prioritized for PSG and early management to prevent the negative consequences, potentially damaging to long-term health [55].

Atopic predisposition is a controversial risk factor for developing OSAS. In a cross-sectional study including 3997 Chinese children aged 3–14 years, Guo et al. [61] observed that the incidence of SDB evaluated by PSQ, in children with asthma, eczema, and rhinitis was significantly higher than in non-atopic children. Similarly, in a cross-sectional study including 681 one-year-old children, Kalra et al. [62] found a 15% prevalence of habitual snoring in children born from atopic parents.

Also, air pollutants can promote the onset of OSAS in children and mostly increase its severity [14]. Several pollutants, such as particulate matter, gases (ozone, dioxide sulfur, and dioxide nitrogen), organic compounds (polycyclic aromatic hydrocarbons), and metals (vanadium, nickel, and manganese) were associated with systemic inflammation, especially in the lung and brain [63,64]. The airway cell barrier in children is particularly sensitive, and it is more permeable to air pollutants [14]. In addition, children inhale a greater volume of air per body weight than adults [14]. Therefore, a greater exposure, a longer pollutant presence in the lungs, and a greater negative effect are observed in children than adults, including an increased susceptibility to respiratory infections and asthma development [65,66,67,68,69,70]. Sánchez et al. [14] carried out a cross-sectional study including 564 children aged 5–9 years where they investigated SDB by using the Pediatric Sleep Questionnaire; the authors showed an association of the exposure to moist and cold air, high levels of ozone, and sulfur dioxide with respiratory sleep symptoms. Indoor environmental pollutants also appear to play a role in the development of SDB. Indeed, children with SDB lived in smaller homes than those without SDB. In addition, 53% of children with SDB underwent exposure to secondhand smoke both outside and inside the home compared with children without SDB (37.1%) [14]. Weinstock et al. [24] in 464 children aged 5–9.9 years evaluated the efficacy of AT compared with medical management by using clinical and polysomnographic data from a previous randomized study. The authors observed that children exposed to tobacco smoke had a 20% higher AHI than patients not exposed.

Among risk factors for developing OSAS in school-aged children, low socio-economic status, and living in low-income neighborhoods play an important role [54,71]. Gueye-Ndiaye et al. [54] carried out cross-sectional analyses in 303 children aged 6–12 years enrolled from the Environmental Assessment of Sleep Youth study from 2018 to 2022 to investigate the risk factors for developing SDB. The authors found an association between the exposure to insects indoor and SDB-related symptoms in children living in low-income neighborhoods. A recent study by Gueye-Ndiaye et al. [71], on 453 children, aged between 3 and 12.9 years observed that children with mild SDB living in low-income neighborhoods showed a low SDB–related QoL (10%) and major symptoms related to SDB (23%), compared to children living in more advantageous neighborhoods.

### 3.3. Predictors of OSAS During Adolescence

Obesity has reaching epidemic proportions in recent years [72], and it represents the major risk factor for OSAS in adolescents [68]. Compared to children of normal weight, overweight or obese children have a 24–61% higher risk of developing OSAS [69]. Obesity acts through several mechanisms. Firstly, the presence of adipose tissue promotes pharyngeal collapsibility in the pharyngeal soft tissue and significantly reduces residual functional capacity in the chest wall, facilitating hypoxemia [1]. Secondly, adipocytes can secrete a wide range of pro-inflammatory cytokines such as TNF α, IL-6, plasminogen activator inhibitor (PAI-1), matrix metalloproteinase-9 (MMP 9), monocyte chemoattractant protein-1 (MCP-1), and hormones such as adiponectin and leptin [70]. Increased leptin levels were found in patients with OSAS [73,74]. As in adults, increased levels of pro-inflammatory cytokines were demonstrated also in obese children with OSAS. For example, a prospective, multicenter study was performed by Gileles-Hillel et al. [75] with 204 children between the ages of 4 and 15 years, 75 of whom had OSAS diagnosed at PSG. The authors observed that IL-6, MCP-1, and PAI-1 levels were significantly higher in obese children with OSAS compared to those without OSAS. The reduction in inflammatory cytokine levels in these patients appears to be linked to the resolution of SDB. Kheirandish-Gozal et al. [76] investigated the effects of AT on plasma levels of inflammation markers in 100 obese children aged 4 to 15 years with OSAS diagnosed at PSG. After AT, only one-third of the patients showed normalization of PSG; however, a significant decrease in MCP-1, PAI-1, MMP-9, IL-18, and IL-6 was observed. These results further suggest the pro-inflammatory effects of SDB such as OSAS. Additionally, any increase in BMI above the 50th percentile results in a 12% increased risk of developing OSAS [77]. Katz et al. [78] showed how neck to waist ratio (a body fat distribution index) could be used as a predictive index for OSAS in obese or overweight children and adolescents.

Several studies evaluated the association between BMI and OSAS development [16,79,80]. Scott et al. [79] observed that the BMI z-score was a predictive factor for OSAS, especially in children more than 12 years old, although it was less predictive in younger children. Graw-Panzer et al. [80] found an association between BMI z-score and OSAS severity in children > 7 years of age. Recently, a retrospective study conducted by Dékány et al. [16] observed a significant association between BMI percentile and AHI in both children under and over 7 years of age.

Obesity and adenotonsillar hypertrophy often coexist. Approximately 45% of obese children have adenotonsillar hypertrophy, and AT is the first-line therapy. However, obesity represents a risk factor for residual OSAS after AT [81]. Indeed, the risk of residual OSAS after treatment is estimated of 33–76% in obese children compared with 15–37% in non-obese children [82].

Regarding the effects of body weight reduction on OSAS, Andersen et al. [83] carried out a prospective study including obese or overweight children with OSAS and observed a normalization of the AHI after 1 year in 44% of children who followed an obesity treatment protocol and showed a significant reduction in BMI. However, most of those patients enrolled (71%) had mild OSAS.

Another risk factor for OSAS in adolescents is chronic nasal obstruction caused by allergic rhinitis [11]. AR is present in 35% of children with snoring and 6% with OSA [84]. Rhinitis affects sleep through several mechanisms. Nasal congestion secondary to the inflammatory process of the nasal mucosa can cause mouth breathing, sleep interruption, and respiratory fatigue [85]. The tone of the nasal mucosa vessels regulates the patency of the nasal cavities, and rhinitis leads to an increased nasal resistance resulting in mucosal edema and the production of mucous secretions [86]. Moreover, an increased airflow velocity may cause restriction of the nasal valves, worsening the airflow obstruction [1]. Additionally, chronic inflammation in the nasal and pharyngeal mucosa could contribute to the onset of SDB promoting tonsil and adenoid hypertrophy. Significant associations were reported between allergic rhinitis and snoring in school and pre-school age groups [52]. Guo et al. [61] found a prevalence of 24.4% of SDB in children with rhinitis compared to 10.7% in children without rhinitis.

In a recent study, Yang et al. [87] evaluated the factors associated with the development and progression of severe OSAS in children. They observed a significant association of male gender and allergic rhinitis with moderate-severe OSAS in a cohort of 263 children with mild (51.3%) and (48.7%) moderate-severe OSAS. The incidence of mild and moderate-severe hypoxemia in this population was 39.2% and 60.8%, respectively, and the presence of AR was the only significant predictor of hypoxemia. In support of these studies, a meta-analysis by Cao et al. [88] with 44 studies and 6086 adult and pediatric participants evaluated the prevalence of AR in patients with SDB and OSA. The authors observed a prevalence of AR of 40.8% and 45.2% in children with SDB and OSA, respectively, with a prevalence of AR in children with SDB 2.12 times higher compared to children without SDB. In addition, a number of inflammatory mediators in rhinitis such as histamine, cysteinyl leukotrienes, IL-1β, IL-4, IL-10, and bradykinin can cause an alteration in sleep architecture in patients with OSAS [89,90]. Histamine is a relevant modulator of the sleep–wake cycle and also regulates mechanisms related to arousal and cognitive and memory function [90]. Krouse et al. [91] found that levels of IL-1β, IL-4, and IL-10 were increased in adult patients with allergic rhinitis compared with those patients without allergic rhinitis and correlated with a reduced latency to sleep onset, reduced latency to REM sleep, and a shorter REM phase duration. Obesity and AR are the predominant risk factors in the adolescent population. However, predictor factors specific to other age groups such as ATH, predisposition to atopy, infections, as well as exposure to environmental pollutants may contribute to the development of OSAS.

## 4. The Bidirectional Link: Asthma and OSAS in Children

The pathophysiological connection of asthma and OSAS represents a complex clinical challenge, as each condition potentially exacerbates the other [92]. Asthma is recognized not only as a risk factor for the development of OSAS, but also as a factor that contributes to its severity, creating a vicious cycle of mutual exacerbation that significantly impacts pediatric patients [93]. Children suffering from both conditions are prone to sleep issues, characterized by difficulty staying asleep and frequent awakenings during the night, resulting in daytime fatigue and weariness [94]. To date, several studies [85,95,96] support the “one-airway” hypothesis, which suggests a dynamic interaction between asthma and OSAS, driven by shared inflammatory mechanisms. This hypothesis states that inflammatory processes that start in the upper airway can worsen inflammation in the lower airway, and vice versa [97].

Moreover, the coexistence of upper and lower airway inflammation, often associated with atopy, can lead to heightened leukotriene responses, which in turn contribute to airway narrowing, increased nasal resistance, and a predisposition of pharyngeal collapse [97,98,99,100]. Long-standing inflammation associated with asthma can adversely affect respiratory muscle function, particularly the muscles responsible for opening the upper airway, increasing the risk of developing OSAS [54]. Acknowledging the coexistence of these diseases is critical, necessitating specific diagnostic procedures such as polysomnography, spirometry or pulmonary function tests to tailor appropriate treatment strategies in these children [101]. Regarding the treatment of children with both diseases, the initiation of Continuous Positive Airway Pressure therapy is more common in patients with concomitant asthma and OSAS compared to those with only OSAS [102]. Leukotrienes, pivotal in the pathogenesis of both asthma [103] and OSAS [104] could represent a therapeutic target. Indeed, leukotriene levels in adenotonsillar tissue are increased in children with OSAS. In recent clinical trials, the administration of montelukast in children with OSAS led to a reduction in adenotonsillar hypertrophy, improved airway patency, and a decrease in apneic events [105]. However, the efficacy of leukotriene pathway modulation in managing pediatric SDB warrants further investigation, potentially reshaping the treatment landscape for children with OSAS [106]. 

Risk and protective factors and likely associated pathophysiological mechanisms are showed in Table 1.

## 5. Limitations

This narrative review has several methodological concerns that need addressing. Firstly, a narrative review is often more subjective, relying heavily on the authors’ expertise and perspective, which can introduce bias in the selection and interpretation of studies. Secondly, the methodology is generally less rigorous, lacking a standardized protocol for searching, selecting, and appraising the literature. Lastly, due to the lack of a systematic approach, it is often less reproducible, with different reviewers potentially arriving at different conclusions based on the same body of literature [110]. However, this review provides a broad and flexible overview of the topic, identifying research gaps and informing future studies.

## 6. Conclusions

In conclusion, our review underscores the dynamic nature of the risk and protective factors influencing OSAS in children, across the different age groups. Pre-school children exhibit a heightened vulnerability to OSAS in case of ATH and exposure to tobacco smoke, whereas obesity emerges as a predominant risk factor in older children and adolescents. Protective factors also display age-related variability, with breastfeeding and nasal steroid use showing greater protective effects against OSAS in younger children. However, studies investigating which protective factors contribute to the development of OSAS are scarce. These findings highlight the need to adopt age-specific strategies in both the prevention and management of OSAS in pediatric populations. Early identification and mitigation of these risk factors, coupled with reinforcement of protective behaviors, could significantly reduce the prevalence and severity of OSAS among children. Consequently, healthcare providers should consider the age of the child when assessing risk and implementing preventive measures for OSAS, ensuring a tailored approach that maximizes effectiveness and patient well-being. Furthermore, artificial intelligence systems seem to be a promising strategy to diagnose OSA. Particularly, the use of clinical characteristics and questionnaires as input could be beneficial in children with suspected OSA to simplify the diagnostic process.

## Figures and Tables

**Table 1:** Risk and protective factors and likely associated pathophysiological mechanisms.

Risk Factors	Age	Pathophysiological Mechanisms
Adenotonsillar hypertrophy	Preschool age Childhood	Upper airway obstruction especially in the retropalatal area.
Obesity	Childhood Adolescence	Adipose tissue accumulated in soft tissues promotes pharyngeal collapsibility and reduces RFC of the lungs.
Rhinitis	Childhood Adolescence	Congestion with increased nasal resistance leading to hypertrophy of tonsils and adenoids.
Infants born prematurely	Pregnancy Preschool age	- Peculiar craniofacial features. - Increased need for invasive therapeutic maneuvers that may alter palate shape and airway growth. - Hypotonia.
Infective factors (RSV)	<2 years of age	RSV can induce neuro-immunomodulatory changes leading to adenotonsillar hypertrophy.
Male	Pregnancy	- Increased airway length in post-pubertal males. - Neurochemical control mechanisms and the response to arousal may vary based on sex.
African American	Pregnancy	- Different airway anatomy. - Differences in neuromotor control of the pharynx. - Other genetic or environmental factors not yet known.
Genetic diseases	Pregnancy <2 years of age	Characteristic craniofacial malformations and typical mechanisms involving the soft tissues of the upper airways promoting the development of OSAS.
Genetic polymorphisms (ApoE4, TNFα-308G, and NADP+)	Pregnancy <2 years of age	- ApoE4: alters membrane stability and has been associated with children with OSAS displaying reduced neurocognitive performance [107]. - TNFα-308G: alterations related to slow-wave sleep [108]. - NADP+: children with OSAS displaying cognitive dysfunction [109].
Craniofacial abnormalities	Pregnancy, <2 years of age	Alterations in the size, geometry, and position of the tongue and jaw can lead to airway narrowing [4,23].
Neuromuscular disorders	Pregnancy <2 years of age	- Reduced ventilatory response. - Reduced activity of respiratory muscles during sleep. - Reduced tone of upper airway dilators. - Poor lung mechanical activity. - Dysfunction of the breath control centers [46].
Air pollutants	Childhood	Increased exposure to, and longer duration of, pollutants in the lungs with increased negative effects of air pollution due to the immaturity of the epithelial barrier and the large volume of inhaled air relative to body weight.
Protective factors	Age	Pathophysiological mechanisms.
Maternal age	Pregnancy	Maternal age appears to be inversely proportional to risk of developing SDB.
Maternal breastfeeding	Pregnancy <2 years of age	- Breastfeeding promotes the formation of a healthy jaw, thus preventing the onset of many of these anatomical problems such as a high palate, narrow dental arches, and a retruded chin [38]. - Breast milk also contains antibodies and other protective factors that help the baby fight infection, thus reducing exposure to viral infectious agents leading to upper airway remodeling such as adenotonsillar hypetrophy [38].

NADP+: nicotinamide adenine dinucleotide phosphate; RSV: respiratory sincizial virus; ApoE4: apolipoprotein E4; TNFα-308G: tumor necrosis factor α-308G.
